# Endoplasmic Reticulum Stress Pathway, the Unfolded Protein Response, Modulates Immune Function in the Tumor Microenvironment to Impact Tumor Progression and Therapeutic Response

**DOI:** 10.3390/ijms21010169

**Published:** 2019-12-25

**Authors:** Manuel U. Ramirez, Salvador R. Hernandez, David R. Soto-Pantoja, Katherine L. Cook

**Affiliations:** 1 Department of Physiology and Pharmacology, Wake Forest University Health Sciences, Winston-Salem, NC 27157, USA; 2 Digital Illustrator, Winston Salem, NC 27103, USA; SalvadorHernandez87@gmail.com; 3 Department of Surgery, Wake Forest School of Medicine, Winston-Salem, NC 27157, USA; dsotopan@wakehealth.edu; 4 Department of Cancer Biology, Wake Forest University Health Sciences, Winston Salem, NC 27157, USA

**Keywords:** unfolded protein response, Inositol-requiring enzyme 1 (IRE1), PKR-like endoplasmic reticulum kinase (PERK), Glucose-regulated protein 78 (GRP78), Activating transcription factor 6 (ATF6), immune cells, T cell, macrophage, tumor microenvironment

## Abstract

Despite advances in cancer therapy, several persistent issues remain. These include cancer recurrence, effective targeting of aggressive or therapy-resistant cancers, and selective treatments for transformed cells. This review evaluates the current findings and highlights the potential of targeting the unfolded protein response to treat cancer. The unfolded protein response, an evolutionarily conserved pathway in all eukaryotes, is initiated in response to misfolded proteins accumulating within the lumen of the endoplasmic reticulum. This pathway is initially cytoprotective, allowing cells to survive stressful events; however, prolonged activation of the unfolded protein response also activates apoptotic responses. This balance is key in successful mammalian immune response and inducing cell death in malignant cells. We discuss how the unfolded protein response affects cancer progression, survival, and immune response to cancer cells. The literature shows that targeting the unfolded protein response as a monotherapy or in combination with chemotherapy or immunotherapies increases the efficacy of these drugs; however, systemic unfolded protein response targeting may yield deleterious effects on immune cell function and should be taken into consideration. The material in this review shows the promise of both approaches, each of which merits further research.

## 1. Introduction

For a cell to become cancerous, it must overcome several evolutionary obstacles [1]. Among these, a cancerous cell must proliferate readily and avoid immune destruction [2,3]. Achieving this state is complex. While mutations to tumor suppressors and proto-oncogenes contribute to these changes, over the past few decades it has become obvious that cancer cells also repurpose several endogenous survival systems to assist in their formation and progression.

We must consider both the tumor cells and their microenvironment to understand how tumor–host interactions drive transformation and carcinogenesis, and subvert these survival systems. The tumor microenvironment has been extensively investigated (reviewed in [1,4,5]). Of note are the tumor immune infiltrates, metabolite availability, and stress effects of the tumor microenvironment. Tumors have been referred to as “wounds that never heal” [6]. Healing mechanisms associated with normal tissue injury promote tumor formation and metastasis. Immune infiltrating cells induce wound-like inflammation in the tumor microenvironment, further assisting in the development of malignant cancers. Poor perfusion of solid tumors leads to high levels of hypoxia and low metabolite availability, and the healing response responds to these via angiogenesis [7,8]. Leaky vasculature results in high osmolarity in the tumor microenvironment. Recent studies have even demonstrated that microbial flora in cancers differ from normal tissues, and these enhance tumor progression [9].

The combination of these factors creates a ‘perfect storm’ to subvert evolutionary pathways and repurpose them to be procancer. One such system of interest is the unfolded protein response (UPR). The UPR is an evolutionarily conserved mechanism discovered somewhat serendipitously [10]. In the mid-1970s studies found that virally transformed cells increased the expression of protein p78. Unknowingly, Hass and Wabl identified the same protein in 1983 [11]. This protein was localized to the endoplasmic reticulum (ER) and bound unsecreted Ig heavy chains, thus named binding immunoglobulin protein (BiP). In 1987, Lee et al. reported that p78 expression in highly proliferative transformed cells was due to media glucose depletion [12]. The protein was named glucose-regulated protein 78 (GRP78). This protein was also identified as heat shock 70 kDa protein 5 (HSPA5). Additional research led to the discovery that BiP and GRP78 were the same protein, and bound to unfolded or incomplete Ig intermediates, identifying GRP78 as the first ER chaperone protein. GRP78 has since become recognized as the primary regulator of the UPR. The UPR is triggered by accumulation of unfolded proteins in the ER, conditions that are often found in highly proliferative, secretory, or pathogen-infected cells [13].

Recent evidence indicates that the UPR is critical in multiple systems, such as cell differentiation, proliferation, immune response, and cell maintenance. This review focuses on the role of the UPR in tumor microenvironment stress, its effect on cancer cell progression, and immune response to cancer cells.

## 2. Body

### 2.1. ER Stress and UPR Signaling

When stressed, the ER is overwhelmed with an accumulation of proteins due to improper folding, insufficient glycosylation, and/or inhibited transport [14]. These often arise due to a sudden increase in protein expression leading to insufficient chaperone proteins, saturation of the ER lumen space, or insufficient nutrients for post-translational modification. These situations are often found in both immune and cancer cells. This accumulation and subsequent ER stress results in canonical UPR signaling through three different proteins: IRE1, PERK, and ATF6.

Each of the three proteins initiate an ‘arm’ of UPR signaling and are thought to be regulated, in part, by association with ER membrane-bound GRP78. Current understanding of this system suggests that, under non-stressed conditions, IRE1, PERK, and ATF6 are primarily bound to GRP78, maintaining an inactive state and preventing UPR signaling. While it does not directly assist in folding, GRP78 binds unfolded proteins to maintain them in a foldable state. When unfolded proteins accumulate, i.e., induction of ‘ER stress’, GRP78 releases IRE1, PERK, and ATF6, preferentially binding unfolded polypeptide chains. This release allows each of these three proteins to initiate their portion of the UPR. The pathway and function of these arms and their effects are briefly described below.

IRE1: Inositol-requiring enzyme 1, also known as endoplasmic reticulum to nucleus signaling 1 (ERN1). IRE1 oligomerizes and autophosphorylates upon release from GRP78. Phosphorylation activates an endonuclease domain that cleaves an intron from the X-box binding protein 1 (*XBP-1*) mRNA. This cleaved mRNA is then translated to the transcription factor XBP-1s. It is unclear whether cleavage occurs in the ER or the nucleus, as IRE1 has been found in the inner nuclear envelope [15]. XBP-1s induces the expression of ER chaperone proteins and ER-associated protein degradation (ERAD) proteins, and induces differentiation of metabolic regulators. XBP-1s also induces *XBP-1* transcription in the form of self-regulation. In addition to effects mediated by XBP-1s, IRE1 continues nuclease function in the ER, degrading ribosomal-associated mRNA through regulated IRE1-dependent decay (RIDD). This degradation prevents the translation and further accumulation of unfolded proteins. IRE1 also contains a kinase function, which phosphorylates c-Jun N-terminal Kinase (JNK), contributing to apoptosis under prolonged UPR signaling [16]. Although GRP78 association is the primary inhibitor of IRE1 activation, there is evidence for alternate methods of IRE1 activation, including direct binding by unfolded proteins [17].

PERK: Protein kinase R (PKR)-like endoplasmic reticulum kinase, or eukaryotic translation initiation factor 2-alpha kinase 3 (EIF2AK3). Release from GRP78 suppression induces PERK oligomerization and transphosphorylation similar to IRE1. PERK then phosphorylates eukaryotic translation initiating factor 2A (eIF2α), preventing the formation of ribosomal pre-initiation complexes and reducing cap-dependent protein translation. An open reading frame in the 5’-untranslated region of activating transcription factor 4 (*ATF4*) mRNA allows cap-independent translation during eIF2α phosphorylation. PERK activity thus increases *ATF4* function to propagate UPR signaling. ATF4 expression results in products enhancing metabolic changes and ERAD, in concert with transcription products from XBP-1s activity. Prolonged UPR leads to cell cycle arrest and, under certain conditions, apoptosis via CCAAT-enhancer-binding protein homologous protein (CHOP) expression downstream of PERK activation. Independent of PERK-mediated ATF4 expression, PERK activation results in an antioxidant response via nuclear factor erythroid 2-related factor 2 (NRF2)-induced expression of genes containing antioxidant response elements (AREs) in their promoters [18].

ATF6: Activating transcription factor 6. ATF6 translocates to the Golgi complex upon GRP78 release. Golgi-localized site-1 and site-2 proteases (S1P and S2P) then cleave ATF6, releasing a cytosolic basic leucine zipper (bZIP) domain. This bZIP domain translocates to the nucleus and induces the transcription of ER chaperones, lipid biosynthesis, and ERAD proteins. These allow expansion of the ER, reducing the density of unfolded proteins and increasing chaperone protein availability, further assisting with reducing the unfolded protein burden and ER stress. Additionally, like XBP-1s, ATF6 induces XBP-1 expression for UPR autoregulation. Prolonged ATF6 activation also leads to a form of CHOP-independent apoptosis.

**Figure 1:**
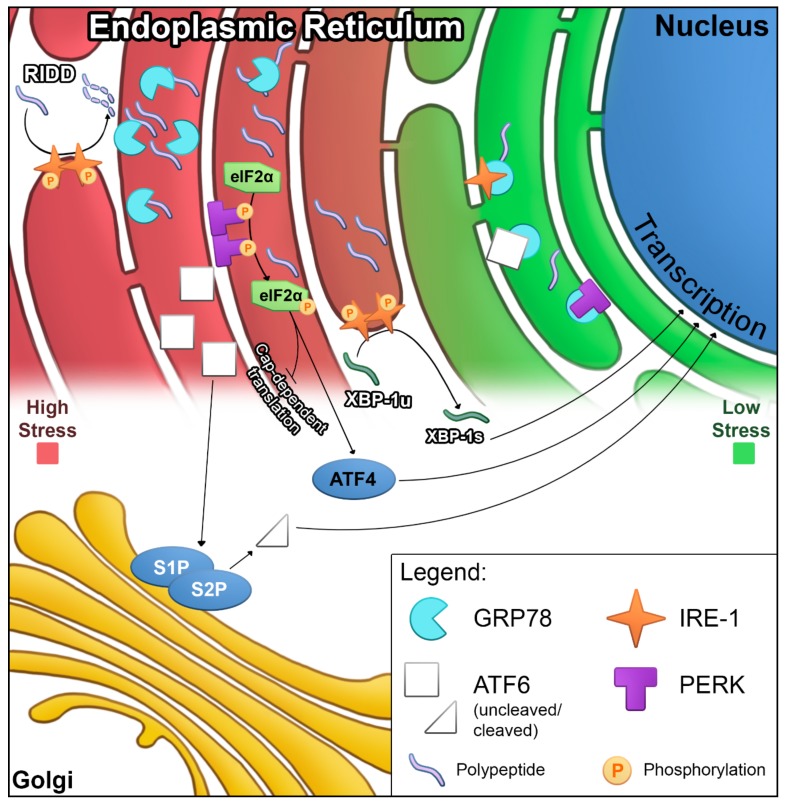
ER-stress induced UPR signaling. Summary mapping of the UPR signaling pathways and locations in which they occur. Each of the three ‘arms’ of UPR signaling are bound by inhibition due to GRP78 sequestration (right, green ER). Under ER stress, GRP78 binds unfolded proteins, releasing IRE1, ATF6, and PERK (left, red ER).

For this review, we will be focusing on UPR signaling through IRE1, PERK, and ATF6 (Figure 1). This is a simplified model of UPR signaling, omitting numerous additional proteins involved in glycosylation, folding, and quality control. IRE1, PERK, and ATF6 signaling pathways work together to reduce ER burden. While this traditional role of UPR is widely agreed upon, recent research suggests that this model requires further refinement and may not be applicable in all cell types, particularly in immune and cancer cells, both of which have atypical expression needs.

### 2.2. ER Stress and the UPR in the Tumor Microenvironment

UPR signaling is frequently upregulated in the tumor microenvironment due to inflammatory factors, the high metabolic rate of cancer cells, elevated hypoxia, and poor nutrient availability. In prostate cancer, tumor cells induce an UPR in the local microenvironment, termed Transmissible ER Stress (TERS), leading to an UPR in neighboring cells [19]. What secreted factors are responsible for TERS are unclear, though TERS appears to be dependent upon Toll-like Receptor 4 (TLR4) activation [20]. It is likely that transmissible ER stress will be found in other cancers as well.

**Figure 2:**
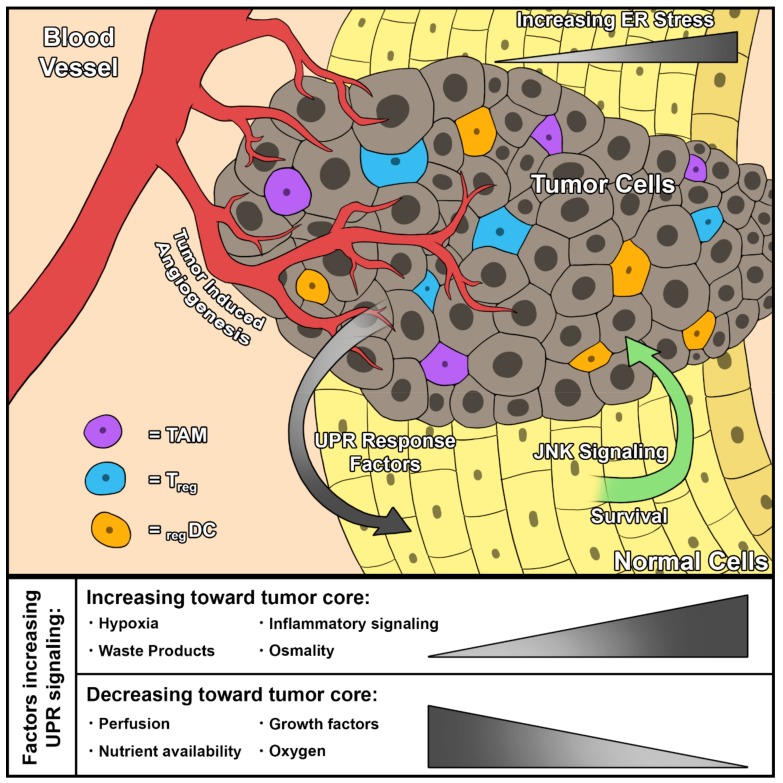
UPR signaling in a tumor and the surrounding microenvironment. The composition of the tumor and its microenvironment promote UPR signaling in cancer cells via various mechanisms. Poor perfusion, low oxygen, and reduced growth factor availability contribute to increased UPR signaling. The ability of cancer cells to exert ‘transmissible UPR signaling’ to the surrounding normal tissue has been associated with increased survival via JNK signaling. TAM, tumor associated macrophage; Treg, regulatory T-cell; regDC, regulatory dendritic cell; JNK, c-Jun N-terminal kinase.

Much like inflammation, UPR in the tumor microenvironment increases tumorgenicity and is associated with a stem-like phenotype, proliferation, angiogenesis, and survival during starvation or hypoxic conditions [21,22,23,24,25,26,27] (reviewed in Figure 2). Increased UPR in neighboring tissues supports tumor development via Wnt signaling. Wnt signaling reduces pro-apoptotic UPR signaling in prostate cancer cells [19]. The UPR may further assist in metastasis of circulating cancer cells to hypoxic regions. UPR signaling is increased in bone metastases of breast, lung, and prostate cancers [28,29,30]. However, the role of the UPR is not clear; there are also reports that UPR activation, through increased ER stress, can induce immunogenic or apoptotic cancer cell death [22,31,32,33]. Simultaneously, reducing UPR signaling can induce immunogenicity and clearance of cancer cells [34,35,36]. There is a balance to UPR signaling that allows cancer progression, without activating cell death pathways.

The role of UPR signaling in various cancers has been a topic of interest for many years, though parsing the exact role of UPR signaling has been difficult. For example, activation of the UPR is reported to prevent apoptosis in prostate cancer cells [37,38], but in another report, UPR was downregulated in murine models of prostate cancer [39]. Interestingly, the arms of UPR signaling had divergent effects in androgen-dependent prostate cancer; IRE1α activity and *XBP-1* expression were increased, but PERK activation was reduced [40]. UPR activation in the microenvironment also induces resistance to bortezomib and paclitaxel in prostate cancer cells [19]. Although UPR activity has a clear role in prostate cancer progression, the subtleties of its activation and how they affect survival are not well understood. Investigating the characteristics of the UPR in prostate and colorectal cancers may still yield effective therapeutic targets [41].

Our group and others have shown breast cancers, that overexpress GRP78 in response to chemotherapies, exhibit resistance to said chemotherapies [35,42,43,44], resistance to anti-estrogen therapies [13,45], and increased tumor anti-immunity [35,36]. This overexpression response is associated with hypoxic [44] and triple-negative [46] breast cancers. UPR-induced resistance may be downstream of MYC (cellular Myelocytomatosis; or *c-Myc*) [26], though *MYC* activation is unlikely to be the only contributor. Expression of ATF6α is correlated with resistance to chemotherapy and reduced time to breast cancer reoccurrence [47]. UPR stress aids age-related breast tumor development, and overcoming estrogen receptor-positive status is associated with UPR induction [48]. Recently, Ypt-interacting protein 1A (Yip1A) regulation of IRE1 and PERK signaling facilitated survival in cervical cancer cells [49].

Although the intricacies of UPR signaling in cancer microenvironment have not yet been deciphered, there is a clear trend that optimal levels of UPR signaling confer immune protection and may assist in the progression of cancer. Due to these findings, investigation of UPR targeting in cancer therapies is of particular interest.

### 2.3. Tumor Immunology

Among the many challenges of cancer formation is immune escape. Mutations in cancer cells lead to increased antigen presentation which stimulates an immune response. Indeed, tumors arise with greater frequency and grow more rapidly in immunodeficient models [50,51]. To overcome this, cancer cells must maintain presentation as ‘self’ or have sufficient immunosuppressive capability in their microenvironment; often, a combination of both. A model used to describe how cancer cells can overcome immune system destruction is ‘immunoediting’. In this model, the immune system effectively prevents tumor development, most often by destruction of tumor cells. In some cases, the immune system can only delay tumor cell growth. This stasis, rather than destruction, allows time for further mutations and resistance to occur, thus selecting for tumor cells resistant to the immune system [50,52]. This model gives rise to an interesting parallel between cancer cells developing immunoresistance and microbes developing antibiotic resistance.

To overwhelm immune response to novel and mutated antigens, cancer cells can simply overexpress ‘self’ markers, inactivating cytotoxic cells [53,54,55]. Beyond identifying as ‘self’, cancer cells can subvert immune system effects to promote growth. While the immune system can induce cancer cell death and quiescence [56,57,58,59,60], it can also promote tumor development and establish a favorable tumor microenvironment [57,61,62,63,64,65,66,67,68]. Which of these effects occur is highly dependent on the microenvironment, what immune infiltrates are present, and the cancer type [69,70,71,72,73,74]. Infiltrates to consider are antigen-presenting cells (APCs), including tumor-invading macrophages (TAMs), neutrophils (TANs), and dendritic cells (DCs). In specific contexts, these cells are associated with survival in multiple cancer types [58,59,60,68,75,76]; however, they can also promote cancer progression in different contexts.

A characteristic of the tumor microenvironment strongly associated with tumor progression and inhibition of therapeutic efficacy is inflammation [63,64,77]. Inflammatory cell infiltrates, including macrophage subtypes, regulatory T-cells (Tregs), neutrophils, and myeloid-derived suppressor cells (MDSCs), create a microenvironment that suppresses cytotoxic cell activity [72,78,79,80,81,82,83,84,85,86]. Counterintuitively, many of these inflammatory infiltrates are recruited by B and T cells in efforts to increase immune response, but instead promote tumor development [83,84,87].

Beyond immunosuppression, the inflammatory environment generated by these immune cells assists in establishing a metastatic niche, inducing cancer cell “stemness” [88]. These cells can induce stemness by multiple means, including adjusting metabolism and further enhancing immunosuppression and tolerance in tumor microenvironment [61,79,82,84,85,86,89,90]. This state includes many features associated with undifferentiated cells, inducing proliferative potential, survival and durability advantages, migratory potential via epithelial-to-mesenchymal transition (EMT), and inducing angiogenesis of solid tumors [74,81]. Increased ‘stemness’ is additionally associated with increased metastatic potential, more malignant cancers, and poorer patient outcomes [91].

Another aspect that must be considered is the function of UPR signaling in tumor immunology. In the context of a highly stressful tumor microenvironment, the function of UPR signaling in individual immune cell types is worth consideration (summarized in Table 1).

### 2.4. UPR Signaling in Dendritic (Myeloid-Derived Suppressor) Cells

Myeloid-derived suppressor cells (DCs) are key regulators in immune cell response [92]. Once mature, DCs are tissue-imbedded and function as APCs. DCs additionally release cytokines to recruit and/or activate other immune cells. DCs regulate T-cell activation through a combination of presenting foreign and dead cell antigens. These steps are important in immune cells recognizing which pathogens and mutated cells must be cleared, and preventing an autoimmune response to healthy self-cells [93].

The ability of DCs to induce immune response requires maturation, presentation of antigens, and cytokine expression/release. Successful maturation of DCs is dependent upon IRE1α activation and *XBP-1* splicing. Inhibition of IRE1 function or abrogation of XBP-1S signaling reduces successful maturation and increases apoptotic death in DCs [94]. High mobility group box 1 protein (HMGB1), known to instigate DC maturation, induces GRP78 expression and XBP-1 signaling [95]. DCs that do mature under these conditions fail to stimulate T-cell proliferation due to decreased CD80, CD86, and major histocompatibility complex (MHC)-II expression and cytokine secretion [94,95].

Inhibition of UPR signaling in matured DCs also reduces their ability to activate T cells. CD80, CD86, MHC-II, and cytokine secretion are all reduced in XBP-1-inhibited mature DCs [95,96]. Inhibition of XBP-1 signaling may prevent cytokine expression and antigen presentation due to compensatory RIDD hyperactivity [97]. IRE1α/XBP-1 signaling is prioritized in DC function and it is generally believed that the PERK/ATF6 arms of the UPR do not significantly contribute to DC function [97]. The inhibition of CHOP, however, specifically prevents the expression of the inflammatory cytokine, interleukin-23 (IL-23) [96]. These findings suggest that the role of UPR signaling in antigen-presenting DCs may be more complex than currently appreciated.

Two additional classes of DCs are functionally defective DCs, which do not successfully migrate or present antigens, and regulatory DCs (regDCs), which are immunosuppressive (reviewed in [98]). Unlike APC DCs, regDCs are associated with progression in cancers and poorer prognosis [99,100]. Tumor-supporting regDCs were reported as early as 1997 [101]. In that study, Enk et al. reported that tumor-suppressive APC DCs converted to immunosuppressive regDCs. RegDCs exist in normal tissues to prevent a hyperactive immune response, along with other forms of regulatory or alternatively activated immune cells (discussed further below). Much like normal tissues, tumor-infiltrating DCs can be ‘reprogrammed’ into regDCs by the tumor microenvironment [102].

DCs in the tumor microenvironment can affect responsiveness or tolerance to therapies [101]. In one study, DCs were isolated from patients exhibiting metastatic melanomas, of which some metastases were responsive to chemotherapy and some metastases remained progressive. In vitro, the DCs isolated from responsive metastases activated T lymphocytes five-fold higher than DCs from progressive metastases, suggesting that the latter exhibited a regulatory DC phenotype and induced chemotherapeutic resistance. Another group showed that regulatory DCs promote metastatic expansion of pancreatic ductal adenocarcinoma [103]. Metastatic sites were enriched in regulatory DCs (CD11b^+^CD11c^+^MHC-II^+^CD24^+^CD64^low^F4/80^low^) that produced Treg cells via secretion of programmed death ligand 2 (PD-L2), a T-cell checkpoint inhibitor ligand. This study also showed the depletion of regulatory DCs, blockade of PD-L2-reduced expansion, and metastatic pancreatic ductal adenocarcinoma in vivo. The role of the UPR in regulatory DCs has yet to be determined; it may play a key role in their formation. Given the role of regulatory DCs in tumor progression and immunotherapy resistance, this area requires further investigation.

### 2.5. UPR Signaling in Macrophages

Macrophages are another class of APC immune cells that regulate the innate immune response. Macrophages rival DCs in antigen presentation to and activation of T cells. This is characteristic of M1 (‘killer’ or ‘classically activated’) macrophages, which induce inflammation and anti-antigen responses. M1 macrophages form from circulating monocytes that infiltrate tissues in response to chemoattractants. M1 macrophages then release inflammatory factors; phagocytize pathogens, cell debris, and unhealthy cells; and present antigens to activate T cells. In contrast, M2 (‘repair’ or ‘alternatively activated’) macrophages suppress inflammatory responses and inhibit T-cell activation. In these contexts, macrophages need to induce sufficient immune response without deleterious effects to host tissues. Poor regulation of this process, or loss of M1 and M2 macrophage function, is associated with progression or initiation of several diseases, including cancers [103].

Macrophages were found to initiate UPR signaling upon differentiation [104]. This study examined both peripheral blood and atherosclerotic-infiltrating macrophages, the latter are comparable to tumor-infiltrating macrophages. Although initiation timing was unclear, GRP78 expression was increased and *XBP-1* transcripts were primarily found in their spliced forms upon monocyte infiltration and macrophage differentiation [104]. GRP78 heterozygous macrophages can differentiate and mature, but expend more energy and have reduced capacity for inflammation [105]. Spliced *XBP-1* expression is also associated with survival in macrophages, potentially through UPR regulating macrophage metabolism via induced autophagy [106]. Importantly, UPR signaling is required to maintain the ratio of inflammatory M1 to suppressive M2 cells via ATF4 expression [51].

Macrophage-induced inflammatory response is associated with IRE-1 in models of arthritis, while data in microglia (neurological tissue equivalent of macrophages) show that PERK activation is required [107,108]. The UPR is further necessary for successful macrophage response under induced ER stress. Chemically induced ER stress prevents macrophage function, but upregulation of PERK/ATF4 signaling can compensate for this effect [109].

Loss of UPR function in macrophages is strongly correlated with disease, including fibrosis [110], obesity-induced inflammatory disease [111,112], tuberculosis [113], and fatty liver disease [114]. These studies associate macrophage-induced inflammation via UPR signaling in these diseases, such that inhibition of UPR signaling abrogated or ablated disease.

Atherosclerosis, narrowing of arteries by plaque lesions, is a macrophage-associated disease in which the role of UPR signaling has been extensively studied (reviewed in [115]). In atherosclerosis, M1 macrophages are recruited to arterial plaques, whereupon they accumulate lipids, becoming foam cells. Foam cells accumulate free cholesterol due to the presence of low-density lipoprotein (LDL), which then induces UPR signaling [116,117,118]. UPR signaling induces CD36 expression, increasing the uptake of oxidized LDL and leading to further upregulation of all UPR signaling arms [119]. PERK activation leads to Glycogen Synthase Kinase 3 Alpha/Beta (GSK3a/b) signaling, further inducing lipid accumulation [120]. These pathways lead to a macrophage–foam cell–UPR self-potentiating cycle. Perpetuated UPR signaling leads to foam cell death via the CHOP pathway [113].

The changes found in atherosclerosis plaques are similar to those in the tumor microenvironment. Fittingly, macrophages have been associated with regulation and immune response in the cancer microenvironment. Increased M1 macrophages are associated with clearance and good prognosis, while M2 macrophages are immunosuppressive and procarcinogenic. Our lab has shown that UPR signaling mediates lipid metabolism in breast cancer, and as a result, macrophages infiltrate into breast cancers [35]. Reducing whole body GRP78 levels by antisense morpholino injection increased macrophage infiltration of breast tumors and reduced the expression of CD47 (“do not eat me”/“self”) signaling in tumor samples. These results were replicated with the administration of linoleic acid, the polyunsaturated fatty lipid cleared downstream of GRP78 activation. Our lab has also demonstrated that inhibition of PERK, but not GRP78 or IRE1 inhibition, is responsible for the increased proliferation of M1 macrophages and cancer cell clearance in melanoma [36]. This study showed that cancer cell UPR activity regulates macrophage response. The inhibition of GRP78 or IRE1 in cancer cells increased macrophage-mediated clearance. The effects of UPR signaling in cancer cells, and their ability to induce UPR signaling in the microenvironment, doubly inhibit macrophage-mediated immune response to cancers.

### 2.6. UPR Signaling in T Cells

T cells are lymphocytes that participate in the adaptive immune response. The T-cell family includes various lymphocytes that mature in the thymus. Each of these cell types contribute to an immune/apoptotic (CD4^+^ helper, CD8^+^ killer, memory, or natural killer) or immunosuppressive (regulatory) role. In proper orchestration, T cells and the APCs that activate them induce an effective response against invading pathogens and malignant cells. Dysregulation of T cells can lead to a compromised immune system or autoimmune diseases.

The UPR is required for successful T-cell formation and activation. Interestingly, different lymphocyte classes exhibit distinct patterns of UPR signaling during differentiation. UPR signaling is activated upon differentiation of both B and T cells. In the presence of a differentiation stimulus, both B and T cells increase GRP78 protein levels, initiate *XBP-1* transcript cleavage, and induce ATF6 signaling [121,122,123,124]. The inhibition of GRP78, ATF6, or XBP-1 signaling pathways greatly reduces plasma cell differentiation and efficacy upon maturation [121,125]. Cell fate determines whether UPR signaling is maintained. For example, early B-cells exhibit UPR signaling, but it is absent in mature B-cells. Similarly, CD4^−^/CD8^−^ progenitor T-cells do not exhibit an UPR, but greatly increase UPR during maturation as CD4^+^/CD8^+^ T-cells. Upon differentiation to CD4^+^ T-cells, the UPR is once again repressed [122].

Unlike B cells and CD4^+^ T-cells, mature CD8^+^ T-cells maintain UPR signaling [122]. XBP-1 signaling downstream of IRE1 is increased during acute infection, and inhibition of XBP-1 signaling prevents terminal differentiation and immune response in CD8^+^ T-cells [123]. T-cell trafficking and homing under oxidative stress also requires UPR signaling [126]. Inhibited signaling, specifically via the GRP78, ATF6, and XBP-1 pathways, greatly reduces plasma cell differentiation and efficacy upon maturation [121,125].

Another area in which the UPR plays a role is T-cell exhaustion [127]. This is a state in which sufficient stimulation does not induce T-cell activation, and thus, the T cell will not proliferate and/or generate the cytolytic compounds required for inducing targeted cell death [128]. The causes for this abnormality may be varied. We do know that a lack of appropriate metabolites and inhibitory signals contribute to this exhausted phenotype. T-cell exhaustion is a concern in numerous diseases, including cancers [127,129,130,131,132]. As stated previously, the microenvironment is hostile and frequently features hypoxia, low metabolite availability, inflammation, and transmissible ER stress responses. All these factors induce UPR signaling, which is directly associated with T-cell exhaustion in models of infectious disease [123,128,133]. T-cell exhaustion in the tumor microenvironment has become an area of interest and potential immunotherapeutic target [127,129,130,132,134].

The specific roles of UPR signaling in T-cell differentiation and activity are incompletely understood. Similarly, the role of UPR signaling in the microenvironment and during activation of helper T-cells has yet to be investigated.

### 2.7. UPR Signaling and Cancer-Associated Fibroblasts

Cancer-associated fibroblasts (CAFs) play a significant role in the development, protection, and metastasis of cancers [135]. CAFs are known to regulate tumor-associated immune cells and warrant mention [136]. Recent findings suggest that UPR signaling plays a large role in the generation of the tumor environment, including the differentiation of CAFs [137]. In turn, CAFs have been shown to stimulate non-small-cell lung cancer invasion by upregulating GRP78 expression [138]. As a regulator of the tumor microenvironment and immune cells, the role of the UPR in CAFs and their function should be further investigated.

### 2.8. Implications for UPR-Targeting Drugs in Cancer Therapy

Interestingly, the efficacy of some chemotherapeutic agents may be due to previously unknown effects on UPR signaling. Triptolide activates UPR signaling (IRE1 and PERK) in breast cancer, inducing cell death, and simultaneously reduces the expression of GRP78. This may be characteristic of several chemotherapeutic agents, for example, nemorosone and ONC212 in pancreatic cancer and nelfinavir in ovarian cancer [139,140,141].

The associations among the UPR, development of clinically diagnosed cancer, and chemotherapeutic resistance have increased interest in targeting the UPR as a strategy for cancer therapy [142]. There is a delicate balance between surviving ER stress and UPR-initiated apoptosis in cancer cells [143]. Disrupting this balance via UPR inhibition [47,48,142,144,145,146,147,148] induces cell death via apoptotic means or immunogenic clearance. Conversely, the overstimulation of the IRE1 and PERK/CHOP pathways [37,149,150,151,152,153,154,155] effectively induces cancer cell apoptosis, likely through pro-apoptotic effects of CHOP. An indirectly activation of UPR signaling by inducing the generation of reactive oxygen species via small molecule therapy leads to cancer cell death in xenografts [153].

Altering UPR signaling may resensitize cancers to chemotherapeutic agents and may increase the efficacy of as yet unknown chemotherapeutic agents. To date, several studies have shown that disrupting UPR signaling increases drug sensitivity. These include reports of abrogating UPR signaling with concurrent drug treatment in murine xenografts [156] and in vivo colorectal cancer models [157,158], and resensitizing breast cancer cells to chemotherapy and immunotherapy [35,36,43,45]. Alternatively, inducing UPR signaling sensitizes non-small-cell lung cancer to doxorubicin [159], instigates ovarian cancer cell death when paired with mifepristone [160], and increases the efficacy of viral antineoplastic therapies [161]. Inducing the UPR was shown to sensitize ovarian cancer cells to chemotherapy via increased JNK signaling [162], which may implicate the IRE1 signaling arm.

There is a growing interest in immunotherapies for cancer treatment. The immune system regularly clears mutated and senesced cells from the body. The goal of immunotherapy is to re-enable the immune system to recognize and clear cancer cells. In general, accomplishing this goal means that immunotherapy must alter cancer cells to present antigens resulting in clearance, or prevent immunosuppressive effects exhibited by cancers. The latter generally focuses on “self” markers expressed by cancers, including cytotoxic T-lymphocyte-associated protein 4 (CTLA-4), programmed cell death protein 1 (PD-1), and lymphocyte activation gene 3 (LAG3). These are checkpoint inhibitor proteins. When immune cell–cancer cell interactions engage these receptors, immune cells cannot begin to activate or proliferate. Our lab has demonstrated that targeting the UPR could induce immune response both through increasing antigen presentation and preventing immune cell inhibition. Targeting GRP78 induces the accumulation of immunogenic polyunsaturated lipids in breast cancer models, inducing macrophage infiltration and clearance [35]. Resistance to (CTLA-4) immunotherapy was also associated with increased UPR signaling [36]. Peripheral blood mononuclear cells (PBMCs) of melanoma patients were collected prior to and post-development of resistance to CTLA-4 immunotherapy with ipilimumab. Arginase 1 (Arg-1) was increased in resistant PBMCs, indicating a shift from M1 cancer-clearing macrophages to M2 immune-inhibitory macrophages. These PBMCs exhibited increased PERK and IRE-1 expression, suggesting that UPR signaling induced the shift to M2 macrophages and subsequent resistance to immunotherapy.

## 3. Conclusions

While the mechanism is still incompletely understood, our knowledge in the functions and activity of the unfolded protein response allows us to examine its role in complex contexts [14,163]. Each of the three arms of the UPR—IRE1, PERK, and ATF6—exhibit unique effects dependent upon the cellular context. Immune cells are particularly reliant upon UPR to handle the stress of rapid division and expression of critical proteins. UPR regulates immune function both in induction of pathogen response and inhibition of autoimmunity [164]. Another context in which UPR is of particular interest is tumor development and tumor microenvironment [21,23,29,31,32,39,48,165,166,167,168]. The role of UPR has been investigated in many types of cancer, suggesting that targeting UPR will be a viable strategy regardless of cancer origin and mutations. Indeed, there are numerous studies targeting UPR as a cancer therapy [27,30,37,48,139,140,141,154,156,162,167,169], to increase chemotherapy efficacy [33,34,44,48,141,142,153,159,160,161,165,170,171,172], and to enhance immunotherapy [34,36,53,132,173,174,175,176].

A better understanding of the roles of each UPR arm in cell and organism homeostasis has the potential to increase the understanding of numerous diseases and this requires further investigation. Given the potential of cancer immunotherapy, understanding the function of UPR in each immune cell type and how this affects their response to cancer cells is of particular interest. In addition to immunotherapy, targeting the UPR shows great promise for increasing the selectivity and efficacy of cancer therapy, and may be a key target in overcoming cancer resistance to chemotherapies. By enhancing efficacy as an adjuvant treatment, targeting the UPR may decrease required concentrations of chemotherapies and therefore off-target effects. Similarly, increasing immunogenicity and clearance of cancer cells by targeting the UPR is likely to be effective with minimal side effects. For these reasons, the mechanisms and role of the UPR in cancer cells, immune response, and how to best target these pathways are high priority targets in furthering cancer treatment.

## Figures and Tables

**Table 1:** UPR signaling in immune cells. A summary of how UPR signaling and each arm function in immune cell differentiation, activation, and function. Areas yet to be investigated are marked with “?”.

Immune Cell Type	Cell Sub-Type	General UPR Signaling	UPR Signaling Component			
			GRP78	PERK	ATF6	IRE-1
DCs	DCs	Required for development and functional antigen presentation and cytokine secretion. Inhibition leads to cell death.	Increased expression during maturation downstream of HMGB1 signaling.	PERK considered to have no association; however, CHOP function required for successful IL-23 secretion.	No associations found upon testing.	Increased during maturation downstream HMGB1 signaling; Required for mature function, CD80, CD86, MHC-1, and cytokine secretion.
	Reg DCs	Unknown.	?	?	?	?
Macrophages	Macrophages	Required for trafficking, function, and M1/M2 polarization.	Increased expression in maturation; decreased expression in target cells increases macrophage efficacy.	Required for mature function; Inhibiting PERK increases M1 polarization; ATF4 function associated with M1/M2 macrophage balance; maintains function during stress signaling.	?	XBP-1 splicing increased; function required for inflammatory response; signaling associated with survival.
	Microglia	Required for function.	?	Required for mature function.	?	?
	Foam cell	Induces CD36 expression, positive-feedback cycle in formation and eventual cell death.	?	Increased function leads to GSK3a/b signaling; CHOP function induces cell death.	?	?
T cell	T cell	Required for various stages of differentiation, maturation, activation, and cytotoxic functions; also required for trafficking and homing. Excessive function associated with T-cell exhaustion.	Increased expression during differentiation.	?	Signaling increased during differentiation, function, and immune response.	XBP-1 splicing increased during differentiation.
	T helper	Required for differentiation but inhibited upon maturation.	Increased expression during differentiation.	?	Signaling increased during differentiation.	XBP-1 splicing increased during differentiation.
	Treg	Unknown.	?	?	?	?
